# Possible significance of degeneration and decreased expression of progesterone receptor in postmenopausal uterine leiomyoma

**DOI:** 10.1186/s12905-022-01924-6

**Published:** 2022-08-16

**Authors:** Saki Tanioka, Ryoko Asano, Reina Wakabayashi, Hiroyuki Hayashi, Hiroyuki Shigeta

**Affiliations:** 1 Department of Obstetrics and Gynecology, Yokohama Municipal Citizen’s Hospital, 1-1 Mitsuzawanishimachi, Kanagawa-ku, Yokohama, 221-0855 Japan; 2 Department of Pathology, Yokohama Municipal Citizen’s Hospital, Yokohama, Japan

**Keywords:** Uterine leiomyoma, Menopause, Progesterone receptor, Estrogen receptor, Degeneration

## Abstract

**Background:**

The growth of uterine leiomyomas is dependent on the levels of sex steroid hormones, and they usually shrink after menopause. However, there are cases in which leiomyomas continue to grow and/or surgery is required after menopause. In addition to estrogen, progesterone has recently been implicated in leiomyoma enlargement, but its relevance to postmenopausal leiomyoma remains unknown. Therefore, we investigated whether hormone receptor expression is associated with postmenopausal leiomyoma enlargement and characterized pathological findings of postmenopausal leiomyoma, which have not been clarified yet.

**Methods:**

Nine cases that required total hysterectomy for leiomyomas after menopause were examined. Surgeries were conducted because of pelvic pressure, pelvic pain, suspected malignancy, or growing leiomyoma. Six cases of leiomyomas being incidentally found during total hysterectomy for postmenopausal uterine prolapse, and six patients who underwent hysterectomy for leiomyomas before menopause, were examined as controls. We evaluated the expression of estrogen receptor, progesterone receptor B, and progesterone receptor AB by immunohistochemical staining among the cases. We also analyzed the pathological findings of leiomyomas.

**Results:**

In postmenopausal leiomyomas, the expression of progesterone receptor was higher than that in the adjacent myometrium. Compared with premenopausal leiomyomas, the expression of progesterone receptor decreased. Postmenopausal leiomyomas that required surgery did not show elevated sex steroid hormone receptor expression, compared with the leiomyomas that did not require surgery.

The degeneration frequency of leiomyomas was 92% in the group that underwent surgery for postmenopausal leiomyomas, 65% in the group that underwent surgery for reasons other than the presence of leiomyomas after menopause, and 47% in the group operated for leiomyomas before menopause.

**Conclusions:**

These results suggest that sex steroid hormones are unlikely to be associated with the growth of leiomyomas after menopause. Since leiomyoma degeneration with increased extracellular matrix is likely to occur in postmenopausal women, the degeneration of leiomyomas may be the main mechanism for the growth of postmenopausal leiomyomas.

## Background

Uterine leiomyomas (leiomyomas), also called fibroids or myomas, are the most common form of benign pelvic tumors in women [1]. Although leiomyomas are considered benign, they cause significant morbidity, such as pain, discomfort, and excessive menstrual bleeding. The growth of leiomyomas is sex steroid hormone-dependent. Therefore, leiomyomas generally tend to shrink after menopause due to a decreased hormone levels [2, 3]. However, a few cases of leiomyomas growing in postmenopausal women have been reported, and the features of postmenopausal leiomyomas are poorly documented. Traditionally, estrogen has been considered as the major promoter of leiomyoma growth. However, in recent years, the role of progesterone in the pathogenesis of leiomyomas has attracted attention [4, 5].

There are two predominant progesterone receptor (PR) isoforms: progesterone receptor A (PRA) and progesterone receptor B (PRB). PRB is an activator of progesterone action, whereas PRA acts as a repressor of PRB [6]. To the best of our knowledge, there are no reports on the expression of PRA or PRB in postmenopausal women with leiomyomas. To validate the significance of sex steroid hormones in leiomyomas of postmenopausal women, we examined the expression of hormone receptors, such as PRB, progesterone receptor A and B (PRAB), and estrogen receptor (ER).

## Methods

### Study design

From January 2009 to September 2016, nine postmenopausal patients underwent a simple hysterectomy for uterine leiomyomas at the Yokohama Municipal Citizen’s Hospital, Yokohama, Japan. This study was approved by the Institutional Review Board of Yokohama Municipal Citizen’s Hospital (No. 201611-2, 2104-2). Written informed consent was obtained from all patients. Cases of variants, such as cellular leiomyoma, lipoleiomyoma, and mitotically active leiomyoma were excluded from the study. Nine patients who underwent hysterectomy for leiomyomas were designated as group A. We included six postmenopausal patients who underwent hysterectomy for pelvic organ prolapse and were incidentally diagnosed with leiomyomas (group B) as well as six patients who underwent hysterectomy for leiomyomas before menopause (group C) as controls. Group B was set up as a control group because leiomyoma was not a direct reason for surgery, even if the presence of leiomyoma was identified preoperatively by ultrasound examination.

Menopause was defined as amenorrhea, for more than 12 months from the last menstrual period. None of the patients received hormone replacement therapy or hormone therapy, such as tamoxifen or gonadotropin-releasing hormone (GnRH) agonists. The maximum diameter of the leiomyomas was measured using pelvic magnetic resonance imaging (MRI) before surgery. If the patient did not undergo an MRI, we measured the maximum diameter of the leiomyoma based on macroscopic findings of surgical specimens.

### Immunohistochemical analysis

Surgical pathology specimens were fixed in 10% neutral buffered formalin, processed conventionally, embedded in paraffin, sectioned, and stained with hematoxylin and eosin (H&E). A pathologist diagnosed uterine leiomyomas (including variants and degeneration) by combining the macroscopic and H&E staining findings. Immunohistochemistry was performed for the ER, PRAB, and PRB expression. The primary antibodies used were as follows: anti-ER rabbit monoclonal antibody (mAb) (SP1) (Nichirei Biosciences, Tokyo, Japan), anti-PRA/B (D8Q2J) XP® rabbit mAb (1:1000, Cell Signaling Technology, Danvers, MA, USA, #8757), and anti-progesterone receptor B (C1A2) rabbit mAb (1:800, Cell Signaling Technology, #3157). Staining was performed according to the manufacturer’s protocol. The staining of ER and PRAB used a detection antibody (anti-rabbit immunoglobulin IgG-linked horseradish peroxidase) and chromogen (3,3'-Diaminobenzidine substrate). The PRB was stained using VECTASTAIN elite ABC Kit, Peroxidase (Rabbit IgG) (Vector Laboratories, Burlingame, CA, USA, PK-6101) and Nova RED Substrate Kit (Vector Laboratories, SK-4800). The signals were scored using the Allred score [7], which is used to estimate the proportion and average intensity of hormone receptor-positive cells in breast cancer as illustrated in Table 1. We evaluated the expression of leiomyoma hormone receptors in groups A, B, and C. Furthermore, we examined the differential hormone receptor expression in the adjacent myometrium and leiomyoma in postmenopausal women (groups A and B).

**Table 1:** Explanation of Allred score

	Observation	Score
**Proportion score (PS)**		
Proportion of positive cells	None	0
	< 1/100	1
	1/100–1/10	2
	1/10–1/3	3
	1/3–2/3	4
	> 2/3	5
**Intensity score (IS)**		
	Negative	0
	Weak	1
	Intermediate	2
	Strong	3
**Total score (TS)**	**PS + IS**	**0, 2–8**

A proportion score (PS) represents the estimated proportion of positive tumor cells (range, 0–5). An intensity score (IS) estimates the average staining intensity of positive tumor cells (range, 0–3). The PS and IS are added to obtain a total score (TS) (range, 0, 2–8)

### Histopathology of leiomyoma

To investigate the histopathology of leiomyomas, we recruited 99 new patients who underwent hysterectomy between March 2019 and February 2020 and created groups a, b, and c with 15 patients of groups A and B. Among the 99 patients, four were postmenopausal patients who underwent surgery for uterine leiomyomas, and along with the nine patients of group A, were included in group a. Group b consisted of 23 cases, with 17 cases in this period and six patients from group B, in which leiomyomas were found incidentally in postmenopausal women. Group c comprised 78 premenopausal patients who underwent hysterectomy and did not receive hormonal therapy before surgery.

### Statistical analysis

All results are presented as medians (interquartile range) or numbers (%) and were compared using the Mann–Whitney test, Kruskal–Wallis test and Fisher’s exact test. Statistical significance was set at *P* < 0.05. Statistical analyses were performed using the IBM SPSS Statistics version 27.0.

## Results

The characteristics of postmenopausal patients (groups A and B) are shown in Table 2.

**Table 2:** Characterization of postmenopausal women who underwent hysterectomy

	Group A: surgery for leiomyoma (n=9)	Group B: surgery for pelvic organ prolapse (n=6)	P-value
Age at surgery	56 (52–69)	61 (54–66)	0.594
Menopause age	50 (50–51)	49 (47.8–52)	0.171
Years passed after menopause	6 (2–19)	11.5 (7–15.5)	0.441
Parity	1 (0.5–2.5)	2 (1.75–2.25)	0.325
Body mass index (BMI)	24.0 (20.4–27.9)	23.3 (18.1–28.1)	0.637
Maximum diameter of leiomyoma (cm)	10.5 (8.5–17.5)	5 (1.8–5.5)	< 0.001
Degeneration	9 (100%)	4 (67%)	0.328
Hyaline	6 (67%)	4 (67%)	1
Edematous	3 (33%)*	0	0.328
Myxoid	2 (22%)	0	0.529

Values are presented as median (interquartile range) or number (%). *Two cases overlapped with hyaline degeneration

The reasons for surgery in group A were as follows: (i) pelvic pressure (four patients), (ii) pelvic pain (three), (iii) suspected malignancy (one), and (iv) no symptoms but the leiomyoma continued to grow (one). The included cases were heterogenous; however, the leiomyomas that required surgeries either tended to grow or did not reduce in size as expected. Surgeries were conducted when both doctors and patients were in agreement. No significant differences were observed in age at surgery, menopausal age, duration after menopause, parity, and body mass index (BMI) between groups A and B. The maximum diameter of the leiomyomas was significantly larger in group A than in group B (10.5 vs 5 cm, *P* < 0.001).

**Fig. 1:**
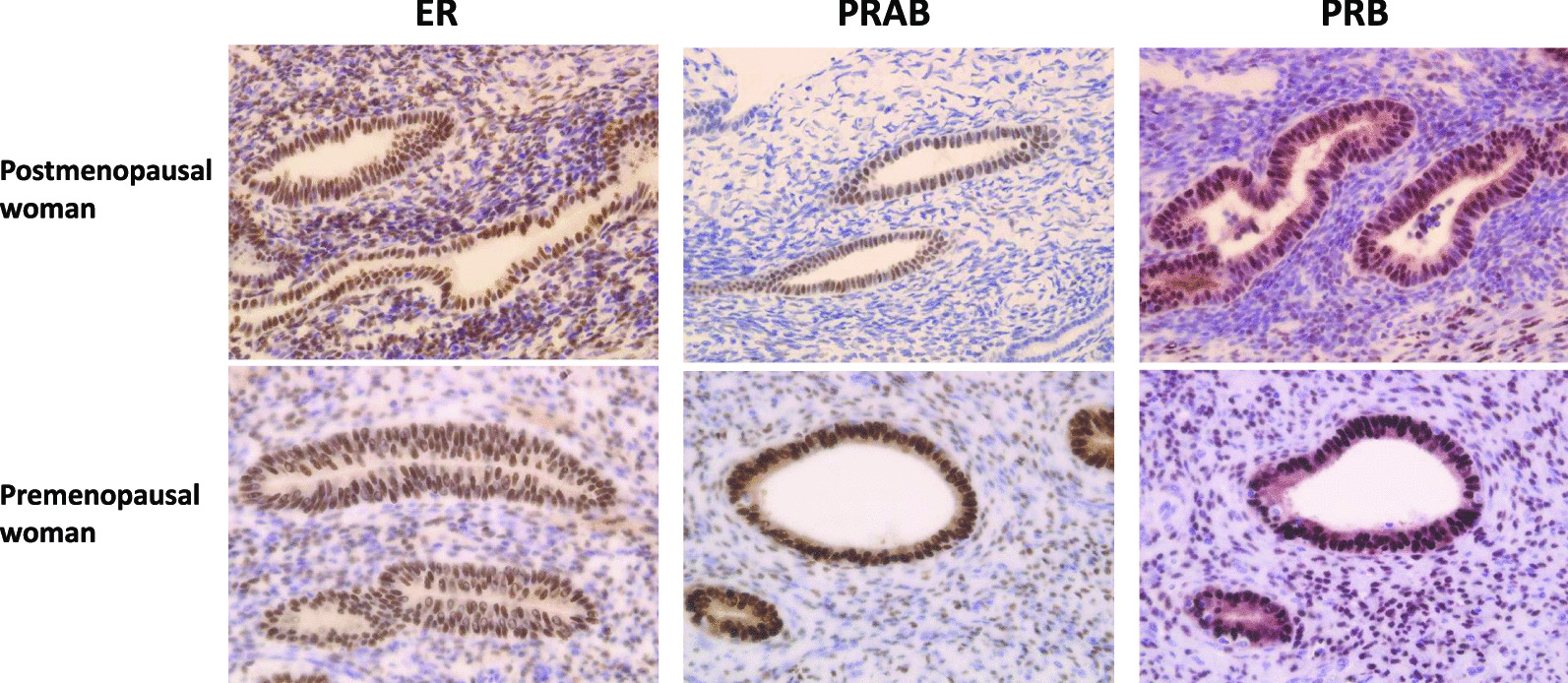
Immunohistochemical staining of ER, PRAB, and PRB in endometria of postmenopausal and premenopausal women. Results for a postmenopausal woman (age: 65 years, 15 years post menopause, underwent hysterectomy for pelvic organ prolapse) and premenopausal woman (age: 43 years) are shown. All slides were stained strongly, with the Allred score being TS 8. ER, estrogen receptor; PRAB, progesterone receptor AB; PRB progesterone receptor B

**Fig. 2:**
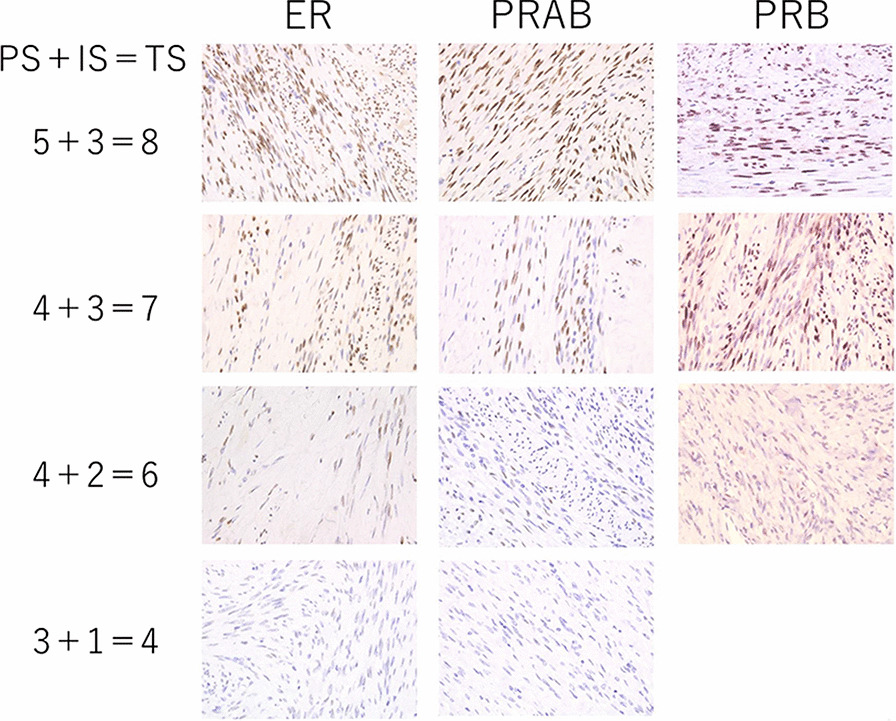
Representative images of slides for each Allred score. PS, proportion score (0–5); IS, intensity score (0–3); TS, total score (0, 2–8); original magnification 400×

**Fig. 3:**
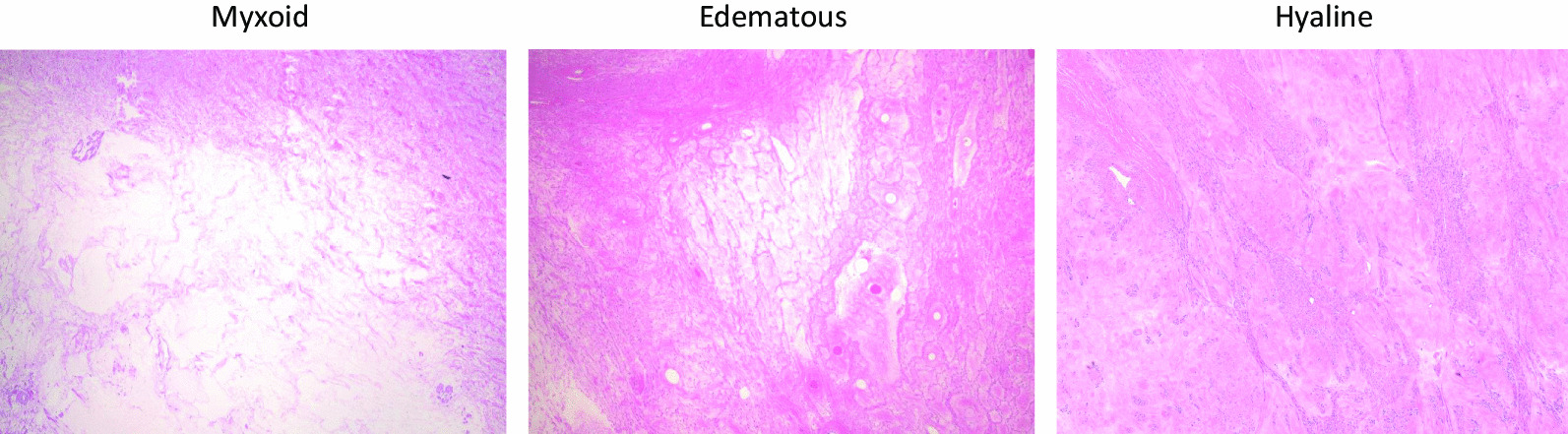
Degeneration images (myxoid, edematous and hyaline) of H&E staining. Original magnification 40×

**Fig. 4:**
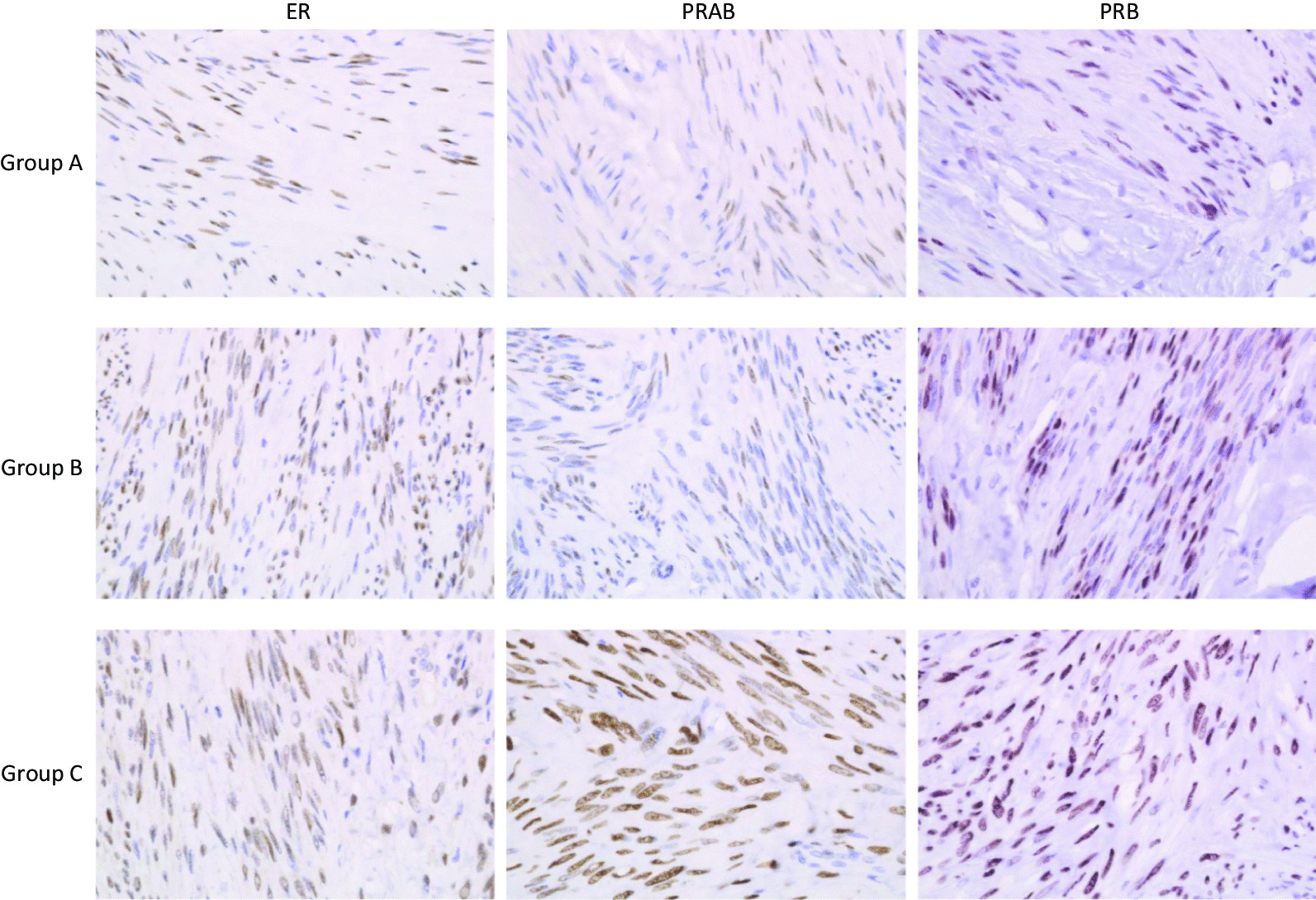
Expression of hormone receptors in leiomyomas of each group. Hormone receptor expression in leiomyomas showed no significant difference in the Allred score of ER, PRAB, and PRB between groups A and B. Groups A and B had lower expression of PRAB compared to that in group C. Group A: postmenopausal women who underwent surgery for leiomyoma; group B: postmenopausal women incidentally complicated with leiomyoma who underwent hysterectomy; group C: premenopausal women who underwent hysterectomy for leiomyoma without hormonal treatment. Original magnification 400×

**Fig. 5:**
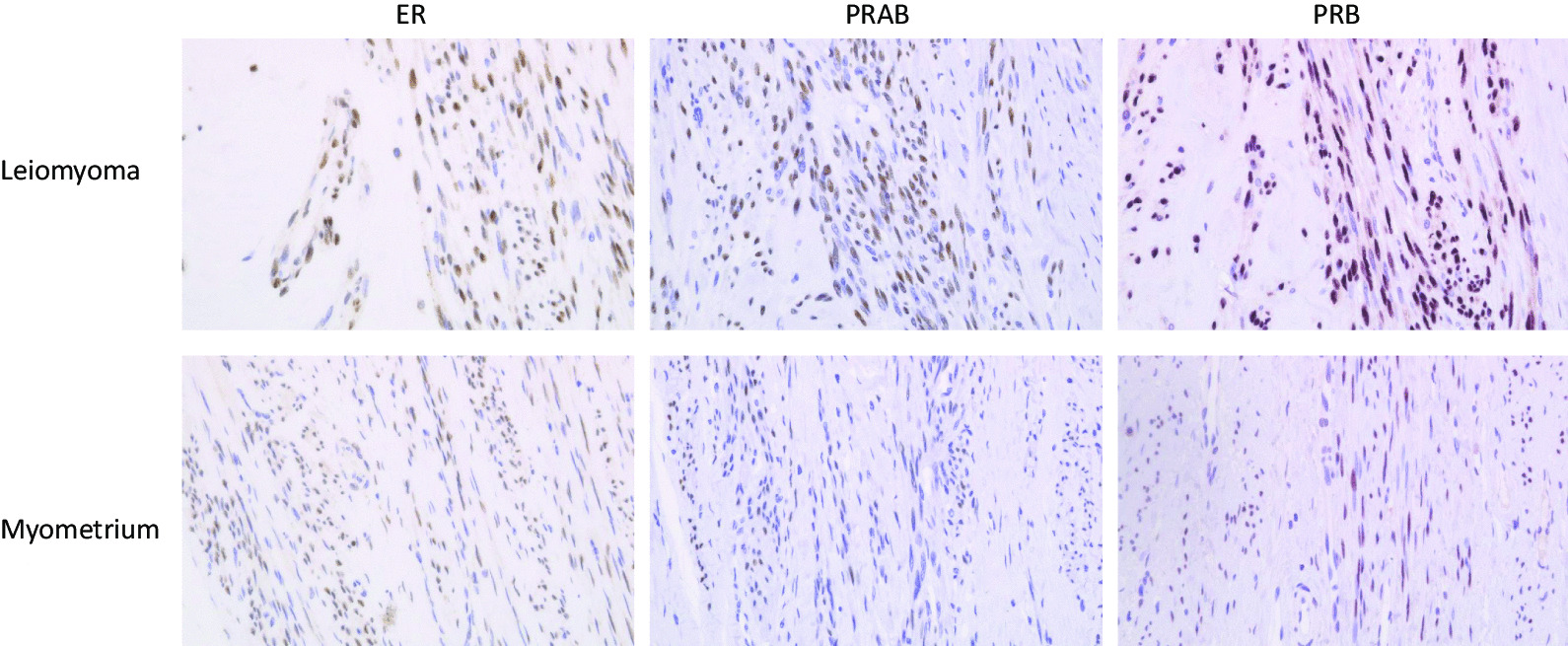
Hormone receptor expression in postmenopausal myometrium and leiomyoma. Expression of PRAB and PRB was significantly higher in leiomyomas than in the adjacent myometrium. Original magnification 400×

We examined the expression of hormone receptors in groups A, B, and C using the Allred score. We confirmed that the expression of all hormone receptors significantly increased in the endometrium even after menopause (Fig. 1). Representative slide glasses for each Allred score are shown in Fig. 2. For group A, clinical and pathological findings and hormone receptor expression levels are shown for each patient (Table 3). Figure 3 shows the prominent areas of degeneration (myxoid, edematous, and hyaline degeneration) on the H&E-stained image. Hormone receptor expressions in leiomyomas between groups A and B showed no significant difference in the Allred score (total score) of ER, PRAB, and PRB (6 vs. 6.5, *P* = 0.543, 6 vs. 6.5, *P* = 0.494 and 7 vs. 7, *P* = 0.896, respectively). Further, we compared postmenopausal and premenopausal cases. PRAB expression in the leiomyomas of postmenopausal patients was lower than that in premenopausal patients (group A vs. group C: 6 vs. 8, *P* = 0.002; group B vs. group C: 6.5 vs. 8, *P* = 0.011). In contrast, the expression of PRB and ER was not significantly different between the leiomyomas of postmenopausal and premenopausal patients. The results of the PRB and ER scores were as follows: group A vs. group C (PRB): 7 vs. 7, *P* = 0.052; group B vs. group C (PRB): 7 vs. 7, *P* = 0.116; group A vs. group C (ER): 6 vs. 7, *P* = 0.145; group B vs. group C (ER): 6.5 vs. 7, *P* = 0.403. Representative images of each group for each receptor expressions are presented in Fig. 4. We further evaluated the expression of hormone receptors in postmenopausal leiomyomas and their adjacent myometrium. When we compared them between leiomyomas and the adjacent myometrium, the Allred score (total score) was 7 vs. 6.5, *P* = 0.259 (ER), 7 vs. 5, *P* = 0.008 (PRAB), and 8 vs. 7, *P* = 0.004 (PRB). The expression of PRAB and PRB was stronger in the leiomyomas than in the adjacent myometrium. Representative images showing the expression of ER, PRAB, and PRB in the leiomyoma and myometrium are presented in Fig. 5.

**Table 3:** List of clinical and pathological findings in group A

Case no	Age	Menopause age	Maximum diameter of leiomyoma	Reason for surgery	ER TS	PRAB TS	PRB TS	Degeneration
1	56	51	17	Suspected malignancy	4	6	7	Myxoid
2	74	50	18	Pelvic pressure	5	4	6	Edematous
3	76	50	10	Continued to grow	7	6	7	Hyaline
4	51	50	9	Pelvic pressure	6	6	6	Myxoid
5	64	50	11.5	Pelvic pain	4	4	6	Hyaline
6	61	54	8	Pelvic pain	4	6	7	Edematous and hyaline
7	51	50	8	Pelvic pain	8	7	8	Hyaline
8	55	49	10.5	Pelvic pressure	7	7	7	Hyaline
9	54	51	25	Pelvic pressure	7	6	7	Edematous and hyaline

Because group A showed a high tendency to have degenerated leiomyomas, we examined the histopathology in group a–c by including degeneration-specific cases. The types of degeneration observed in the pathological diagnosis were hyaline, myxoid, calcification, edematous, fibrosis, and hemorrhage. The results are presented in Table 4. The probability of developing degenerated leiomyomas was 12/13 (92%), 15/23 (65%), and 37/78 (47%) in groups a, b, and c, respectively. There was a significant difference in the ratio of degeneration between groups a and c (*P* = 0.015); however, no significant difference was observed between groups a and b (*P* = 0.407). We focused on myxoid and edematous, cross-tabulated with or without either finding, and performed a Fisher's exact test, which showed that group a had a significantly more degenerative findings than group b (*P* = 0.002). (The number of patients with myxoid or edematous degeneration was seven in group a and one in group b.)

**Table 4:** Frequency of leiomyoma degeneration in postmenopausal and premenopausal women

	Group a (n = 13)	Group b (n = 23)	Group c (n = 78)
Age (years)	58 (54–70)	69 (65–76)	45.5 (43–48)
Maximum diameter of leiomyoma (cm)	11.5 (9–17)	3.0 (1.8–5)	7.5 (6–9.7)
Degeneration	12 (92%)	15 (65%)	37 (47%)
Hyaline	7 (53%)	13 (56%)	16 (21%)
Myxoid	3 (23%)	0	1 (1.3%)
Edematous	4 (31%)	1 (4.3%)	12 (15%)
Calcification	0	2 (8.7%)	2 (2.5%)
Fibrosis	0	0	13 (17%)
Hemorrhagic (Red degeneration)	0	0	4 (5.1%)

Values are presented as median (interquartile range) or number (%). Group a: Postmenopausal women who underwent surgery for leiomyoma; group b: Postmenopausal women incidentally complicated with leiomyoma who underwent hysterectomy; group c: Premenopausal women who underwent hysterectomy for leiomyoma without hormonal treatment

## Discussion

Since many uterine leiomyomas (leiomyomas) do not require intervention after menopause, the pathological findings of postmenopausal leiomyomas have not been clarified. Leiomyomas grow and shrink depending on hormone levels. Leiomyomas express more sex steroid hormone receptors than does the normal myometrium [8], and PR expression in leiomyomas declines under GnRH agonist therapy [9]. Therefore, the expression of hormone receptors is considered to be significant in the growth of leiomyomas.

England et al. reported contents of PR in the leiomyoma were lower during GnRH agonist treatment, compared with the proliferative phase whereas ER expression was maintained during GnRH agonist treatment [8]. Another study showed that when xenografts were treated with 17-beta estradiol (E2) and progesterone (P4), the groups not received E2 (P4 alone or no hormone administration) did not express PR, indicating that E2 is necessary for PR expression. In contrast, ER expression was observed in all xenografts regardless of E2 or P4 administration [10]. These reports corroborate with our results that PR expression was lower in postmenopausal leiomyomas than in premenopausal leiomyomas while ER expression was preserved after menopause.

There are two major progesterone receptor isoforms, PRA and PRB, which are transcribed from the same gene by two alternative promoters. PRB acts as a transcriptional activator of progesterone-responsive genes, whereas PRA acts as a ligand-dependent repressor of PRB [11, 12]. Leiomyomas contain more progesterone receptors (both PRA and PRB) than those found in normal uterine muscle cells [13]. Our study showed that PRAB and PRB were overexpressed in uterine leiomyoma tissue compared to their expression in normal myometrial tissue of postmenopausal women.

Approximately 40% of the patients show predominant expression of PRB mRNA in the superficial part of leiomyomas in premenopausal women. The relative overexpression of PRB may be associated with leiomyomas growth [14]. After menopause, serum estrogen and progesterone levels were significantly lower than those observed before menopause; however, we speculated that the difference in the relative expression of PRA and PRB in postmenopausal leiomyoma may contribute to leiomyomas growth. As a result, we found no difference in the expression of sex steroid hormone receptors between postmenopausal leiomyomas that needed surgery and those that did not. These results suggest that sex steroid hormones may be less relevant to leiomyomas growth after menopause. Comparing pre- and postmenopausal leiomyomas, PRB is mildly decreased in postmenopausal leiomyomas despite a marked decrease in PRAB, suggesting that a decrease in PRA may be involved in the pathogenesis of postmenopausal leiomyomas.

We found that almost all surgically treated postmenopausal leiomyomas showed degenerative changes. Some form of degeneration has been reported in 65–66% of postmenopausal leiomyomas [15, 16]. In our study, degenerative changes were found in 92% of postmenopausal leiomyomas requiring surgery and in 65% of postmenopausal asymptomatic leiomyomas and were found in 47% of premenopausal leiomyomas. Degenerative changes in leiomyomas are considered to occur due to inadequate blood supply [17], and are likely to occur in cases of subserous leiomyoma or relatively large leiomyoma [18]. Degenerative changes are also likely to occur when GnRH agonists are used [19] and during pregnancy [20, 21], when there are changes in hormonal dynamics. Leiomyomas rarely grow after menopause; therefore, only a few cases of leiomyomas have been reported in postmenopausal women [22–25]. An age-related decrease in blood supply to the uterus causes circulatory disorders in leiomyomas, resulting in vitrification of the extracellular matrix and accumulation of fluid and gelatin in leiomyomas [25]. In particular, myxoid or edematous degeneration was found in more than half of the leiomyomas that required surgery after menopause; these type of degeneration with increased extracellular matrix, may be one of the mechanisms leading to the growth of leiomyomas after menopause.

The limitation of this study is that we could not get the results of PRA directly, because PRA is a truncated form of PRB. Therefore, the results of PRA were obtained by subtracting the results of PRB from PRAB. Because immunohistochemical scoring was used to evaluate hormone receptor expression, the ratio of PRA to PRB expression could not be evaluated. In addition, because this study was conducted on Japanese women who were not obese, the effect of estrone (E1) from adipose tissue may be small. Therefore, it is possible that different results will be obtained when examined in other races.

## Conclusions

In postmenopausal leiomyomas, PR expression in leiomyomas was stronger than that in normal myometrium; however, it was weaker than in premenopausal leiomyomas. In contrast, no increase in the expression of hormone receptors was observed in postmenopausal leiomyomas that needed to be removed. Because degenerative characteristics are often observed in leiomyomas that tend to grow after menopause, degeneration may be the main mechanism by which leiomyomas grow after menopause.

## Data Availability

The datasets used and analyzed during the current study are available from the corresponding author on reasonable request.

## Abbreviations

E2: 17-Beta estradiol
ER: Estrogen receptor
GnRH: Gonadotropin-releasing hormone
H&E: Hematoxylin and eosin
IS: Intensity score
P4: Progesterone
PR: Progesterone receptor
PRA: Progesterone receptor A
PRAB: Progesterone receptor A and B
PRB: Progesterone receptor B
PS: Proportion score
TS: Total score
